# Effects of Acupuncture on Sensory Perception: A Systematic Review and Meta-Analysis

**DOI:** 10.1371/journal.pone.0113731

**Published:** 2014-12-12

**Authors:** Petra I. Baeumler, Johannes Fleckenstein, Shin Takayama, Michael Simang, Takashi Seki, Dominik Irnich

**Affiliations:** 1 Multidisciplinary Pain Center, Department of Anaesthesiology, University of Munich (LMU), Munich, Germany; 2 Department of Traditional Asian Medicine, Tohoku University, Sendai, Japan; 3 Institute for Medical Information Sciences, Biometry and Epidemiology, University of Munich (LMU), Munich, Germany; Beijing Institute of Radiation Medicine, China

## Abstract

**Background:**

The effect of acupuncture on sensory perception has never been systematically reviewed; although, studies on acupuncture mechanisms are frequently based on the idea that changes in sensory thresholds reflect its effect on the nervous system.

**Methods:**

Pubmed, EMBASE and Scopus were screened for studies investigating the effect of acupuncture on thermal or mechanical detection or pain thresholds in humans published in English or German. A meta-analysis of high quality studies was performed.

**Results:**

Out of 3007 identified articles 85 were included. Sixty five studies showed that acupuncture affects at least one sensory threshold. Most studies assessed the pressure pain threshold of which 80% reported an increase after acupuncture. Significant short- and long-term effects on the pressure pain threshold in pain patients were revealed by two meta-analyses including four and two high quality studies, respectively. In over 60% of studies, acupuncture reduced sensitivity to noxious thermal stimuli, but measuring methods might influence results. Few but consistent data indicate that acupuncture reduces pin-prick like pain but not mechanical detection. Results on thermal detection are heterogeneous. Sensory threshold changes were equally frequent reported after manual acupuncture as after electroacupuncture. Among 48 sham-controlled studies, 25 showed stronger effects on sensory thresholds through verum than through sham acupuncture, but in 9 studies significant threshold changes were also observed after sham acupuncture. Overall, there is a lack of high quality acupuncture studies applying comprehensive assessments of sensory perception.

**Conclusions:**

Our findings indicate that acupuncture affects sensory perception. Results are most compelling for the pressure pain threshold, especially in pain conditions associated with tenderness. Sham acupuncture can also cause such effects. Future studies should incorporate comprehensive, standardized assessments of sensory profiles in order to fully characterize its effect on sensory perception and to explore the predictive value of sensory profiles for the effectiveness of acupuncture.

## Introduction

Acupuncture is gaining popularity as a non-pharmacological option in pain medicine [1], [2]. There is substantial evidence for acupuncture being effective in the treatment of acute [3]–[5] and chronic pain [6]. However, for several other pain conditions such as neuropathic pain [7] or fibromyalgia [8], evidence remains inconclusive. In order to specify indications for which acupuncture should be used and to optimize treatment, it is crucial to understand how the effect of acupuncture is mediated.

Various mechanisms underlying the effect of acupuncture have been suggested. Brain imaging studies have shown that acupuncture alters activation patterns in brain areas associated with pain processing [9]. It is postulated that in response to the needle stimulation mechanisms of the endogenous pain modulation such as diffuse noxious inhibitory controls (DNIC), segmental inhibition, and descending pain control pathways lead to a decrease in pain perception [10], [11]. At this, various centrally and/or peripherally acting neuromodulators and neurotransmitters such as endorphins [12], serotonin [13], ATP [14], etc. have been identified to play an important role in the analgesic effect of acupuncture. In summary, one can assume that a modulation of the nervous system forms a central part of the effect of acupuncture although details are far from being understood. In special, effects on afferent nerve fibers which might be critical to the modulation of sensory perception by acupuncture remains unclear.

For investigating how acupuncture operates through the nervous system assessments of sensory threshold changes are essential. Evaluations of sensory detection and pain thresholds is referred to as Quantitative Sensory Testing (QST) and has been recognized an important tool in basic science, clinical trials, and for diagnostic and monitoring purposes [15]. QST is deemed to allow for inferences about the type of nerve fibers and about the structures of the nervous system that are affected by a disease or an intervention, according to which modality of sensory perception is changed and at which body sites these changes occur [16]–[18].

Despite the extensive use of sensory threshold assessment in acupuncture research, the impact of these data on the understanding of how acupuncture acts on the nervous system has never been systematically analyzed. Yet, there is no consensus about which modalities of sensory perception (thermal and/or mechanical thresholds, detection and/or pain thresholds) are affected by acupuncture, and whether this effect is influenced by other factors e.g. the measurement tool, the type of stimulation or the target population. The aim of this systematic review, therefore, is to give an overview about data available on the effect of acupuncture on sensory thresholds and to substantiate the respective findings by meta-analyses of high quality studies. Our work provides the first summary of knowledge about how sensory perception is modulated by acupuncture which is crucial to approach a better comprehension of its mechanisms and to improve treatment.

## Materials and Methods

The study protocol containing all steps followed for systematically reviewing literature and performing meta-analyses is available from the authors. Reporting was conducted in accordance with the PRISMA statement [19] as depicted in S1 Table.

### Literature Search

Pubmed, EMBASE, and Scopus were searched from their respective inception dates (Pubmed 1948, EMBASE1988, Scopus 1823) to the 1rst of June 2012 using the following search strategy. 1: acupuncture; 2: perception; 3: sensory; 4: threshold; 5: pressure AND pain; 6: pain AND thermal; 7: heat AND pain; 8: cold AND pain; 9: mechanical AND pain; 10: vibration; 11: experimental AND pain 12: experimentally AND pain; 13: #2 OR #3 OR #4 OR #5 OR #6 OR #7 OR #8 OR #9 OR #10 OR #11 OR #12; 14: #1 AND #13

### Selection Criteria

We included research articles published in English or German which describe the effect of manual acupuncture (MA; needle insertion with or without manipulation), electroacupuncture (EA; needle insertion with electrical stimulation), or dry needling (DN; needle insertion into myofascial triggerpoints (MTrPs)) on thermal or mechanical detection or pain thresholds in humans. Animal studies, studies using other types of pain paradigms (e.g. electrical or ischemic pain), and studies investigating acupuncture related techniques (e.g. laser stimulation or transcutaneous electrical nerve stimulation) were excluded.

### Article Selection

In a first screening step, two reviewers (PIB and JF or PIB and ST respectively) independently assessed the articles retrieved from the literature search for relevance by title and abstract. Full texts of all remaining articles were obtained and screened for eligibility by two independent reviewers (PIB and JF). When the reviewers disagreed or had doubts, the full-text paper was evaluated by all authors. Remaining disagreements were resolved via discussion and consensus.

### Quality Assessment

Two independent researchers (PIB, JF) evaluated the quality of the included studies by assessing the risk of bias by means of the Cochrane Collaboration's tool [20] and the quality of reporting of interventions and assessments of sensory thresholds based on the *Revised Standards for Reporting Interventions in Clinical Trials of Acupuncture* (STRICTA) [21]. In brief, risk of bias was assessed by answering questions about the following features of the studies with ‘Yes’ (low risk of bias), ‘No’ (high risk of bias) or ‘Unclear’ (lack of information or uncertainty over the potential for bias): random sequence generation, allocation concealment, blinding of participants, blinding of outcome assessment, incomplete outcome data, selective reporting and other bias. When sham acupuncture interventions were used as controls we assumed that participants were blinded for group allocation. Blinding of therapists was excluded from the risk of bias assessment since it is not possible to keep the acupuncturist unaware of the point location, stimulation and type of needle. Prior to analysis possible sources of ‘other bias’ were determined by consensus of the authors. This included bias due to a short washout phase in cross-over studies, questionable outcome assessment, and large baseline differences which were not taken into account in the subsequent analysis. Studies lacking a control group were a priori rated as having a high risk of bias. In addition, we report the total sample size as well as the number of study participants in each group.

### Data Extraction

Articles were analyzed by two independent researchers (PIB, JF). Information from the included articles was extracted and tabulated.

Eligible studies are described according to the sensory threshold under investigation, the type of needle stimulation, the characteristics of the study population, and whether the immediate or long term effect of acupuncture was studied. The outcome of the studies is rated as positive or negative according to the authors' conclusion regarding pre-post treatment effects or group differences. Articles with elusive data presentation were rated as unclear. In addition, we compared the effects of verum acupuncture to the effects of inactive or sham-control procedures as well as local to distant needling effects (homo- to heterosegmental, ipsi- to contralateral).

### Statistical Analysis - Meta-Analysis

Chi-squared test was used to test whether a positive study outcome (effect of acupuncture on at least one sensory threshold) was independent of the type of needle stimulation (MA or EA).

A meta-analysis was conducted in order to compare verum and sham acupuncture in high quality studies. Studies were eligible for meta-analysis if they fulfilled the following criteria: no rating of ‘high risk of bias’ in none of the items of the Cochrane risk of bias tool, sham-controlled, blinding of the outcome assessment, and clear reporting of data. All studies fulfilling these criteria were grouped according to reviewers' opinion about clinical homogeneity. We were able to combine studies in which the pressure pain threshold (PPT; kPa) had been assessed before and after a series of acupuncture treatments in patients suffering from musculoskeletal pain. One of the selected studies followed a cross over design [22]. Accordingly, we only included data obtained at baseline and after the first treatment phase. In cases of multiple evaluations of the outcome at one time point, e.g. several measure sites, data were averaged in order to achieve an equal weighting of all studies for the analysis. In order to account for baseline differences, we used delta scores (post-treatment values minus pe-treatment values) for all calculations. A conservative estimator of the delta score variance was obtained according to the variance sum law without correcting for dependent samples. Meta-analytical comparison of effects of verum and sham acupuncture on the PPT was performed by using the package 'metafor' from the R-project (Version 2.15.1, www.metafor-project.org). The standardized mean difference (SMD) was calculated by dividing the delta scores of the verum and sham acupuncture group by the pooled standard deviation of the two groups. Cochran's Q test was applied to evaluate statistical heterogeneity (I^2^). We regarded heterogeneity between studies as substantial if Tau^2^ was greater than zero and either I^2^ was greater than 50% or the Cochran's Q test resulted in a low P value (less than 0.10). An assessment of reporting biases did not appear meaningful due to the small number of studies included in the meta-analyses.

## Results

### General Aspects

**Figure 1:**
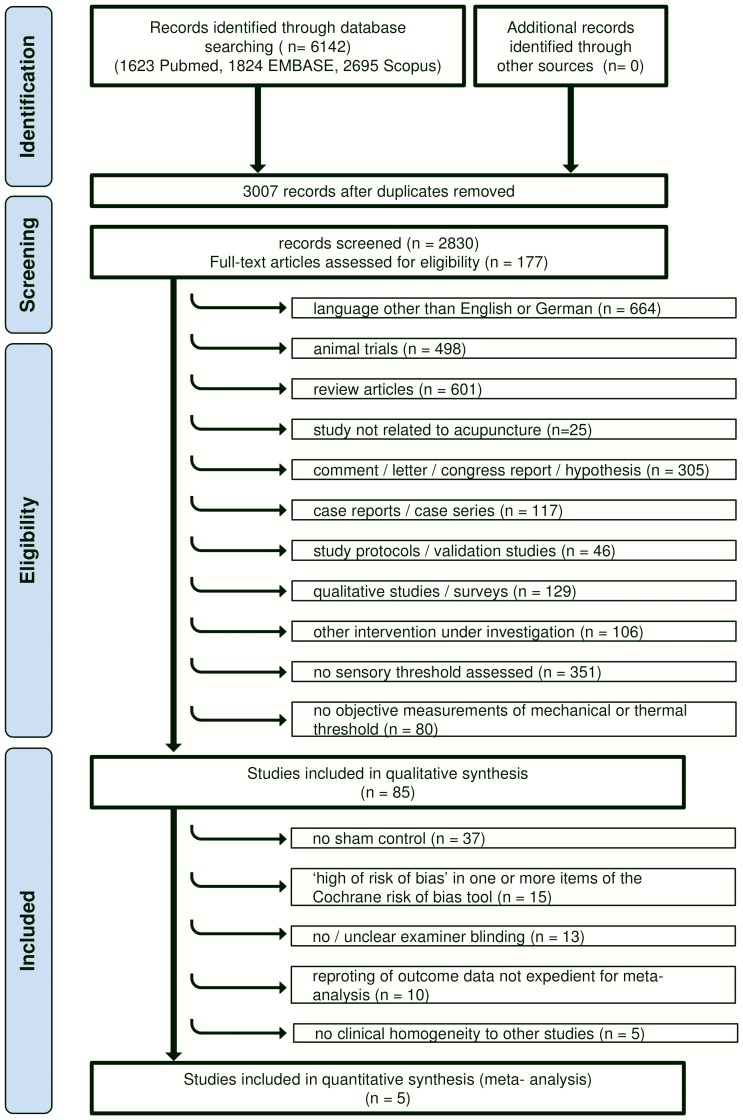
Systematic Review Process Flowchart.

By electronic literature search we identified 3007 citations of which 2922 were excluded; 2830 were screened by title or abstract in a first selection step, and full text was obtained of 177 articles (fig. 1). Eighty five articles published between 1974 and 2012 met our inclusion criteria (table 1 & 2). More than half of these articles (50 out of 85, 58.8%) were issued after 1999.

**Table 1:** Studies Conducted with Healthy Subjects.

Author et al.	Year	Threshold	Verum Interventions	Control Interventions	Acupuncture Points (Verum Treatment)	Measure Site	Threshold
							Changes
Amand [24]	2011	CPT	MA	Non-acu. point stimulation	TH 5, LI 4, SI 4 (dominant side)	Forearm (dominant side)	x
				Period of rest			verum = sham
Anderson [25]	1974	CPT	EA	Non-acu. point stimulation	LI 11, LI 5, SI 5, SI 8 (right)	Hand (bilat.)	x
				Period of rest			Verum > sham
Ashton	1984	CPT	MA	Placebo pill	PC 7 (non-dom. side)	Hand (non-dom.)	x
			TENS 100 Hz				
			TENS 8 Hz				
Barlas [28]	2006	PPT	EA low intensity	Non-penetrating needle	LI 10, TH 5, GB 34, ST 38 (dom. side)	First dorsal interosseous muscle of the hand (bilat.)	x
			EA high intensity	with or without patient blinding			Verum > sham
Benoliel [29]	2011	WDT	MA 4 points	-	1) ST 6, LI 4 (bilat.)	Dermatome of infraorbital nerve & mental nerve (bilat.)	x
			MA 6 points		2) ST 6, LI 4, ST 2 (bilat.)		
Berlin [30]	1975	HPT	EA	Non-acu. point stimulation	LI 4, TH 5 (left)	Post lateral aspect of the left forearm	x
				Period of rest			verum = sham
Brockhaus [31]	1990	HPT	MA	Non-acu. point stimulation	LI 4 (bilat.)	Forearm ventral side (left)	x
			LA	Sham-laser			Verum > sham
Chae [32]	2006	HPT	MA	-	LI 4 non-dom. hand	Distal digit of finger of dom. hand	x
Clark [34]	1974	HPT	EA ipsilat.	-	HT 1, SI 9, HA 3 LU 5, TH 3, LI 4	6 sites on volar surface of forearm (bilat.)	x
			EA contralat.		(body side according to study group)		
Croze	1976	HPT	MA	Non-acu. point stimulation	LI 10, ST 36 (right)	Thenar eminence (left)	unclear
				• close to measure site			
				• distant to measure site			
				Period of rest			
Day [36]	1975	HPT	EA	-	LI 4, GB 21, TH 8, LI 14 (bilat.)	Area over thyroid gland, chest (right)	-
Downs [99]	2005	CDT, WDT	MA	Non-penetrating needle	TH 5, LI 11 (right)	Thenar eminence (right)	-
		CPT, HPT		Period of rest			verum = sham
Farber [39]	1997	PPT	EA	-	LI 4, 1 needle at 5 mm distance for EA	LI 5, LI 11, 15 mm lateral from LI 5 or LI 11,	x
					induction (unilat., side not indicated)	(side not indicated), LI 20 (bilat.)	
Galloon [42]	1977	HPT	EA	Non-acu. point stimulation	not indicated	Forehead, throat, chest, forearm, upper leg (right)	-
			Morphine	Placebo pill			verum = sham
Kitade [55]	1988	HPT	EA + D-Phenylala.	EA + placebo pill	LI 4, LI 10 (bilat.)	Palmar side of forearm (side not indicated)	unclear
Kitade [53]	1979	HPT	7 different EA regimens	Non-acu. point stimulation (auricular)	1) auric. lung (bilat.)	ST 32, KI 23, BL 11, PC 4, GB 35 (bilat.), EX-HN 3	x
				Period of rest	2) auric. sympathicus (bilat.)		(no statistics)
					3) auric. shen-men (bilat.)		Verum > sham
					4) auric. kidney (bilat.)		
					5) auric. neck (bilat.)		
					6) auric. elbow-arm (bilat.)		
					7) auric. lung + LI 4 (bilat.)		
Kitade [54]	1981	HPT	EA + D-Phenylalanine	EA + lactose	LI 4 & ST 36 (bilat.)	Center part of anterior forearm	x
			EA + L-Phenylalanine	D-Phenylalanine			(no statistics)
Knox	1977	CPT	EA	Period of rest	LI 4, EX-UE 2 (right)	Forearm (right)	x
Knox [56]	1981	CPT	EA	Period of rest	LI 4, EX-UE 2 (right)	Forearm (right)	-
			Hypnosis				
Knox [57]	1979	CPT	EA	Non-acu. point min. stimulation	LI 4, TH 5 (unilat., side not indicated)	Hand & forearm (ipsilat.)	x
				Period of rest			Verum > sham
Knox [59]	1977	CPT	EA	Period of rest	LI 4, EX-UE 2 (unilat., side not indicated)	Hand & forearm (ipsilat.)	-
Kong [60]	2005	HPT	EA	Non-penetrating needle	LI 4, ST 36, SP 6 (right)	Forearms & legs (bilat.)	x
			MA				Verum > sham
Kong [61]	2009	HPT	EA	Non-penetrating needle	LI 3, LI 4 (right)	Medial aspect of forearm (right)	x
							verum = sham
Lang [100]	2010	HPT, CPT, WDT, CDT,	MA	-	SP 6, SP 9, ST 36, GB 39 (left)	Anterolateral skin of lower limb	x
		TSL, MDT, MPS, MPT,	EA (80 Hz)			(sensory region of peroneal nerve; bilat.)	
		VDT, PPT	EA (2 Hz)				
Leung [101]	2005	CDT, WDT, CPT, HPT,	EA	-	SP 1, LR 1 (left)	4 points on calf & thigh along spleen & liver meridian (bilat.)	x
Leung [102]	2008	CTD, WDT, CPT,	EA 5 min	-	SP 1, LR 5 (left)	4 points on calf & thigh along spleen & liver meridian (bilat.)	x
		HPT, MDT	EA 15 min				
			EA 30 min				
Li [65]	2008	PPT	4 different MA regimens	-	1) LI 4 (right)	LI 10, GB 20 (right) ST 36, KI 3, LI 5,	x
					2) LI 4 (bilat.)	non-acu. points on the upper limbs (bilat.)	
					3) LI 11 (right)		
					4) LI 11 & LI 4 (right)		
Lim [66]	1977	HPT	5 different EA regimens	Acu. point no stimulation	1) LI 4 (right)	Forearm (bilat.)	x
					2) ST 36, 1) (right)		verum = sham
					3) Ulnar nerve (right)		
					4) Auricular: shenmen, 3) (right)		
					5) LI 4, 4) (right)		
Lin [67]	1981	HPT	2 different MA regimens	-	1) SP 6 (right)	Palm & sole of the foot (bilat.)	x
					2) LI 11 (left)		
Lloyd [69]	1976	HPT	EA	-	2x near elbow, 1x ventral & 2x dorsal part of	Back of the hand (right)	-
					forearm, 2x between thumb & first finger (right)		
Lundeberg [103]	1989	TSL VDT	MA 2 regimens	Acu. point no stimulation (LI 4)	1) ST 7 (bilat.)	TSL: upper lip	-
			EA 2 Hz 2 regimens	Acu. point no stimulation (ST 7)	2) LI 4 (bilat.)	VDT: tip of index finger, forearm, fore head (side not indicated)	verum = sham
			EA 80 Hz 2 regimens				
Lynn [105]	1977	HPT, CPT MPS, MPT	5 different EA regimens	-	1) Auricular: stomach	Thigh, abdomen, neck (side not indicated)	x
					1) ST 36, SP 6		
					2) Auricular: stomach, ST 36, SP 6		
					3) SP 6, SP 9		
					4) LI 4, PC 6 (each bilat.)		
Moret [71]	1991	CPT	EA + placebo pill	EA + Naloxone	LI 4, LI 11 (right)	Hand (right)	x
			Hypnosis + placebo pill	Hypnosis + Naloxone			
Pauser [73]	1975	MPT	EA	Non-acu. point stimulation	PC 6, LI 4, auric. lung (side not indicated)	5 defined points on the neck (side not indicated)	x
							verum = sham
Schliessbach	2011	PPT	EA	Non-penetrating needle	LI 4, LI 11 (unilateral, side not indicated)	2nd toes (ipsilat.)	x
[76]			MA				Verum > sham
Schliessbach	2012	PPT	MA	Non-penetrating needle	LI 4 (unilateral, side not indicated)	2nd toes (ipsilat.)	x
[98]			Ice water				verum = sham
Shukla [80]	2011	HPT	EA	Acu. point min. stimulation	LR 1, SP 1 (left)	Medial calf (left)	x
							Verum > sham
Stern	1977	CPT	EA	Non-acu. point stimulation	LI 4, LI 11, LI 14, LI 15 (left)	Length of fingers (left)	x
							Verum > sham
Stewart [85]	1977	HPT	EA	Non-acu. point stimulation	LI 4, ST 36 (bilat.)	Epigastrium, sternum, upper arm & lower leg (left),	x
				Period of rest		forearm & thigh (right)	Verum > sham
Umino [90]	1984	HPT	EA	-	LI 4, LI11, ST36 (bilat.)	Forearm (side not indicated)	x
Wang [92]	2009	CPT	EA 20 min	Acu. point no stimulation	ST 36, SP 6 (left)	3 sites on lower leg (medial, right)	x
			EA 30 min				Verum > sham
			EA 40 min				
Yoon [94]	1986	HPT	EA	Non-acu. point stimulation	1 individually chosen acu. point (bilat.)	ST 6, LI 20 (bilat.)	x (no statistics)
							Verum > sham
Zaslawski [95]	2003	PPT	MA	Acu. point no stimulation	LI 4 (right)	LI 20, PC 6, SI 3, 2R, LI 10, 1R, LI 5, CV 12, 3R, ST 36,	x
				Non-acu. point stimulation		Non-acu. points on forearm, wrist, & lower limb (right)	Verum > sham
				Non-acu. point no stimulation			
				Sham-laser			
Zhang [97]	2003	CPT	EA	Acu. point min. stimulation	ST 36, SP 6 (left)	Thenar eminence (left)	x
							Verum > sham

Sensory thresholds are abbreviated as follows: CDT: cold detection threshold; CPT: cold pain threshold; HPT: heat pain threshold; MDT: mechanical detection threshold; MPS: mechanical pain sensitivity; MPT: mechanical pain threshold; PPT: pressure pain threshold; TSL: thermal sensory limen; VDT: vibration detection threshold; WDT: warm detection threshold; Acupuncture styles are abbreviated as follows: EA: electroacupuncutre; MA: manual acupuncture; DN: dry needling; Acupuncture points are abbreviated according to the WHO standard international nomenclature [147].

**Table 2:** Studies Conducted with Patients.

Author et al.	Year	Threshold	Verum Interventions	Control Interventions	Treatment	Subjects	Acupuncture Points (Verum Treatment)	Measure Site	Threshold
					Frequency				Changes
Abuaisha [23]	1998	VDT	MA	-	6x within 10w	Diabetic neuropathy	LI 3, SP 6, SP 9, ST 36 (all bilat.)	Great toe (side not indicated)	-
Ahn [106]	2007	WDT	MA (Japan. style)	-	1x/w for 10w	Diabetic neuropathy	Individually chosen according to acupuncture style used	Lower lilmb (bilat.)	unclear
		CDT	MA (TCM)						
Barlas [27]	2000	PPT	MA acu. points	Non-acu. point min. stimul.	1x/d for 5d	Healthy induced DOMS	1) PE 2, LI 11, LU 5, LI 4 (non-dom. side)	Biceps brachii muscle at 8 equidistant points	-
			MA tender points	Period of rest			2) 4 most tender points, of biceps brachii (non-dom. side)	(non-dom. side)	verum = sham
Chou [33]	2011	PPT	DN	Non-penetrating needle	1	Chronic muscle pain	TH 5, LI 11 (ipsilat. to active MTrP)	Active MTrP in upper trapezius muscle	x
			MA						Verum > sham
Deluze [37]	1992	PPT	EA	Non-acu. point min. stimul.	6x within 3w	Fibromyalgia	LI 4, MA 36 (bilat.)	18 tender points	x
									Verum > sham
Edwards [38]	2003	PPT	DN + Stretch	Stretch	Individually	Musculoskeletal pain	Active MTrPs	MTrP treated	x
				Waiting list	over 3w				
Fernandes-	2010	PPT	DN	Acu. point no stimulation	1	Temporomandibular disorder	Most painful MTrP in m. masseter & mandibular condyle	MTrP treated	x
Carnero [40]									verum > sham
Fu [41]	2007	PPT	DN along muscle	-	1	MTrPs in neck	7–8 cm aside from the most painful MTrP in the neck	MTrP treated	x
			DN across muscle						
Goddard [43]	2002	PPT	MA	Non-acu. point stimulation	1	Pain in jaw muscle	LI 4, ST 6 (bilat.)	Sensitive area of m. masseter	x
									verum = sham
Harris [45]	2008	PPT	MA	Non-penetrating needle	9x within 4w	Fibromyalgia	"traditional acupuncture treatment" (not further indicated)	Thumbnail (left)	unclear
Harris [44]	2006	PPT	MA	Acu. point no stimulation	1x/w (week 1–3)	Fibromyalgia	"traditional locations" (not further indicated)	Thumbnail, lateral epicondyle (bilat.)	unclear
				Non-acu. point stimulation	2x/w (week 6–8)				
				Non-acu. point no stimul.	3x/w (week 11–13)				
He [46]	2004	PPT	EA + MA +	Non-acu. point no stimul. +	10x within 3–4w	Chronic neck & shoulder pain	EA: 4 Ex-points (not indicated), GB 21 (bilat.), BL 12,	28 MTrPs bilat. on neck & shoulders	x
			auricular seeds	auricular seeds			DU 14, SI 14, SI 15 (unilat. side not indicated)		Verum > sham
							MA: LI 4, LI 11, GB 31 (bilat.), auricular seeds: shenmen,		
							neck, cervical spine, shoulder, shoulder-joint,		
							shoulder-back (unilat.)		
Hübscher [47]	2008	PPT	MA	Non-acu. point min. stimul.	Immediately,	Healthy induced DOMS	GB 34, LU 3, LU 5, LI 11, SP 10,	7 equidistant points along line joining insertion of	-
				Period of rest	24 h and 48h post		ah shi-points (non-dom. side)	bicepts brachii on radius & acromion	verum = sham
					DOMS induction			(non-dom. side)	
Ilbuldu [48]	2004	PPT	DN	Sham-laser	DN 1x/w for 4w	MTrPs in upper trapezius	3 MTrPs in the upper trapezius on both sides	Not indicated	-
			LA		LA/SL 3x/w for 4w	muscle			verum = sham
Irnich [49]	2001	PPT	MA + DN	Sham-laser	5x within 3w	Chronic neck pain	Individualized MA (mostly SI 3, UB 10, UB 60, LR 3, GB 20,	Levator scapulae, trapezius descendens,	-
			Massage				GB 34, TH 5, auric. neck), DN at MTrPs	paravertebral of 6th cervical spine (bilat.)	verum = sham
Irnich [50]	2003	PPT	MA	Non-acu. point stimul.	3x within 10d	Epicondylopathy	LI 4, LI 10, SI 3, GB 34, TH 5 (unilat. on affected side)	Insertion of common tendon of m. extensor carpi	x
								radialis at lateral elbow, belly of m. extensor	Verum > sham
								carpi radialis brevis at transit between proximal	
								third & distal two thirds of affected forearm	
Itho [51]	2011	PPT	MA seg.3 mm	Period of rest	1	Healthy induced DOMS	1) & 2) max. tender point within m. extensor digitorum	20 mm distal to max. tender point within	x
			MA seg.10 mm				3) max tender point on distal third of belly of tibialis anterior	m. extensor digitorum	
			MA heteroseg. 10 mm						
Karst [52]	2000	PPT	MA	Non-penetrating needle	2x/w for 5 w	Chronic tension-type headache	Obligatory: LI 4, LR 3, GB 20; Optional: GB 8, GB 14, GB	Temporal region where palpation had shown	x
							21, GB 41, UB 2, UB 10, UB 60, LU 7, TH 5, ST 8, ST 36,	anterior part of temporal muscle to be most	Verum > sham
							ST 44, DU 20 (maximum of 15 needles, side not indicated)	prominent (bilat.)	
Kotani [62]	2001	PPT	Press needles at	Press needles at	20x 24 h within 4w	Abdominal scar pain	Painful points in scar area	Painful points treated in scar area	x
			painful points	non-painful points					Verum > sham
				Waiting list					
Kumnerddee [63]	2009	PPT	MA	-	5x within 10d	Myofascial back pain	7 acupuncture points, MTrPs (location not indicated)	All MTrPs	x
			Massage						
Li [64]	1983	PPT	EA affected side	-	1	Syringo-myelia	LI 4, PC 6 (unilat., side acccroding to study group)	TH 5 (bilat.), forehead	x
			EA normal side						
List [68]	1993	PPT	MA + EA	Waiting list	6–8x within 6-8w	Temporomandibular disorder	MA:EX-HN 2, ST 7, ST 6, GB 2 20 EA at LI 4, ST 36	Belly of the right & left masseter muscle	x
			Occlusal splint				(unilat. side not indicated)		
Lundeberg [104]	1988	TSL	MA	Acu. point min. stimulation	1	Sinus pain,	LU 7, LI 4, GB 20, ST 6, ST 7, LI 20, EX-HN 3, EX-HN 5	Face (bilat.),	
		HPT	EA 2 Hz	Placebo TENS		Healthy controls	(painful side)	dorsal aspect of hand on painful side	-
			EA 80 Hz						
Ma [70]	2010	PPT	MA + stretching	Stretching	1–2x within 1–2w	Myofascial pain syndrome	MTrPs in upper trapezius	MTrPs (not further indicated)	x
			Miniscalpel + stretching						
Nabeta [72]	2002	PPT	MA	Non-penetrating needle	1x/w for 3w	Fibromyalgia	All tender points (neck, shoulders, back)	All tender points (neck, shoulders, back)	x
									Verum > sham
Perez-Pa-lomares [74]	2010	PPT	DN	-	1x/w over 3w	Chronic low back pain	8 needles placed bilat. within dermatomes L2 to L5	MTrPs (location not indicated)	x
			EA						(no statistics)
Price [75]	1984	HPT	EA	-	1	Low back pain	1) BL 24, BL 25, BL 26, BL 27	Lower back & volar forearm (of painful side)	x
							2) GB 30+1)		
							3) BL 50, BL 51, BL 57		
							(bilat.; semi-standardized; 3-5 needles)		
Seidel [77]	2002	PPT	MA	Sham-laser	2x/w for 4w	Chronic neck pain	15 acu. points (individually chosen, mostly bilat.)	Not indicated	x
			LA 7 mWatt						verum = sham
			LA 30 mWatt						
Shen [78]	2007	PPT	MA	Non-penetrating needle	1	Healthy + clenching teeth 2min	LI 4 (left)	M. masseter (right)	x
						(DOMS)			Verum > sham
Shen [79]	2009	PPT	MA	Non-penetrating needle	1	Myofascial pain in jaw muscle	LI 4 (left)	M. masseter (right)	x
									Verum > sham
Singh [81]	2006	PPT	MA	-	2x/w for 8w	Fibromyalgia	Semi-stand. alternating anterior (GB 20, GV 14, GB 21, SI	18 tender points (bilat.)	x
							12, GB 30, BL 25, BL 23, BL 40) & posterior points (LR 3,		(no statistics)
							GB 34, KI 25, LI 4, ST 36, SI 11, ST 40, SP 6, LR 8)		
							(side not indicated)		
Sprott [82]	2000	PPT	MA	-	1x/w for 6 w	Fibromyalgia	Individualized	12 tender points (bilat.)	x
Srbley [83]	2010	PPT	DN	Non-acu. point stimulation	1	MTrP in m. supra-spinatus,	MTrP in m. supraspinatus (right)	MTrPs in m. infraspinatus &	x
						m. infra-spinatus &		m. gluteus medius (right)	Verum > sham
						m. gluteus medius			
Takeda [86]	1994	PPT	MA	Non-acu. point min. stimul.	3x/w for 3w	Osteoarthritis of the knee	EX 31, EX 32, SP 9, ST 35, GB 34 (affected knee)	Four points on the affected knee: medial &	x
								lateral joint lines, distal musculo-tendinous	verum = sham
								junctions of m. vastus medialis & lateralis	
Targino [87]	2008	PPT	MA + tricyclic	Tricyclic antidepressants. +	2x/w for 10w	Fibromyalgia	LR 3, LI 4, PC 6, GB 34, SP 6 (bilat.) Ex-HN 3	18 tender points (bilat.)	x
			antidepressants. +	exercise					
			exercise						
Tong [88]	2010	VDT	MA	Acu. point min. stimulation	1x/d for 15d	Diabetic neuropathy	LI 4, ST 40, LI 11, ST 36, SP 6 (bilat.)	Medial malleolus muscle in lower extremities	x
								(side not indicated)	Verum > sham
Ulett [89]	1978	CPT	EA	-	1	Chronic pain	LI 4, LI 11, LI 15, LI 14 (left)	Length of fingers (left)	x
			Hypnosis						
Vincente-Barrrero [91]	2012	PPT	MA	-	1x/d for 3d,	Temporomandibular disorder	EX-HN 5, TH 21, GB 2, TH 17, ST 6, LI 4, ST 36, TH 5,	Preauricular, m. masseter,	x
			Decompression splints						
Xue [93]	2004	PPT	EA	Non-acu. point min. stimul.	4 week phase	Tension type headache	Acu. points individually chosen	Frontal, suboccipital, posterior cervical,	x
								m. masseter & temporalis (bilat.)	Verum > sham
Zhang [96]	2009	PPT	MA: PC7	-	5x/w for 2w	Plantar fasciitis	PC 7 or LI 4 (contralat. to pain or bilat. when bilat. pain)	Medial tubercle of calcaneum of non-painful foot	-
			MA: LI4					& most painful site on painful foot	
Zhu [22]	2002	PPT	MA + EA	Non-acu. point min. stimul.	9x/3w	Chronic neck pain	Individualized: MA at two local points (1 bilat.),	GB 20, GB 21, SI 15, GV 20, Ex-HN 5 (bilat.),	-
							2 distal points & EA at 2 distal points	Go 20	verum = sham

Sensory thresholds are abbreviated as follows: CDT: cold detection threshold; CPT: cold pain threshold; HPT: heat pain threshold; MDT: mechanical detection threshold; MPS: mechanical pain sensitivity; MPT: mechanical pain threshold; PPT: pressure pain threshold; TSL: thermal sensory limen; VDT: vibration detection threshold; WDT: warm detection threshold; Acupuncture styles are abbreviated as follows: EA: electroacupuncutre; MA: manual acupuncture; DN: dry needling; Acupuncture points are abbreviated according to the WHO standard international nomenclature [147].

Most of the studies were performed in the US and Europe (49 out of 85, 57.6%) and were published in English (81 out of 85, 95.3%). The majority (77 out of 85, 90.6%) describe the effect of acupuncture on a single sensory threshold [22]–[98], whereas eight studies (9.4%) assessed acupuncture evoked changes in more than one sensory threshold [99]–[106]. About half of the studies (44 out of 85) were conducted with healthy volunteers and evaluated the immediate effect of one acupuncture session (table 1). Forty-one studies (48.2%) included subjects suffering from pain conditions and assessed either the effect of one single acupuncture treatment (13 out of 41; 31.7%) or a series of treatments (29 out of 41; 70.7%; table 2).

In 65 studies (76.5%) an effect of acupuncture on at least one sensory threshold was observed. A statistically significant effect of acupuncture was found in 60 studies (70.6%). In contrast, 15 studies (17.6%) showed no effect on any sensory threshold. In five of the included publications (5.9%), authors report a pronounced threshold change after acupuncture, but no statistical analysis was provided. Still, we classified the outcome of these studies as positive, in terms of an effect of acupuncture, on the basis of individual reasoning. The overall review results were not affected by these studies and the individual reasoning will be stated in the respective chapter [53], [54], [74], [81], [94]. The results of another five studies (5.9%) were rated as unclear due to poor reporting [35], [55], [106], or because only a pooled analysis of all study arms was reported (table3).

**Table 3:** Overall Study Outcome.

Threshold	Healthy			Patients		
	+	-	?	+	-	?
Heat Pain	18	4	2	1	1	-
Cold Pain	10	6	-	1	-	-
Pressure Pain	7	-	-	27	6	2
Mechanical Pain	3	-	-	-	-	-
Warm Detection	3	2	-	-	-	1
Cold Detection	2	2	-	-	-	1
Thermal Sensory Limen	1	2	-	-	1	-
Mechanical Detection	-	2	-	-	-	-
Vibration Detection	0	2	-	1	1	-

“+” indicates a change of the respective threshold through acupuncture, “-” indicates no effect of acupuncture, “?” indicates an unclear study outcome. Studies in which more than one sensory threshold was assessed are listed several times, respectively.

Forty studies (47.1%) investigated the effect of EA, 41 studies (48.2%) the effect of MA. Both were equally frequent shown to be effective in changing sensory thresholds (EA 32 out of 40, 80.0%; MA 30 out of 41, 73.2%; Chi-squared-test n.s.). In three studies effects of EA tended to be larger than those of MA [60], [76], [100]. DN was evaluated only with regard to its effect on the PPT, which was found to be elevated in six out of seven studies (8.2%). In four studies (4.7%) different types of needle stimulation were combined, with two showing a significant effect and two showing no effect. In six studies (7.1%) acupuncture was combined with another intervention. Among these, five resulted in altered sensory thresholds.

### Quality of Included Studies

Twenty nine out of the 85 included studies (34.1%) were performed with a group size of 20 subjects or more [22], [23],[31],[37],[41],[48]–[50],[52],[56],[57],[62],[64],[65],[68],[74],[76],[81]–[84],[86]–[89],[93],[96],[98],[100], and ten studies (11.8%) did not include a control group [23], [32], [36], [39], [69], [75], [81], [82], [90], [101] (table 4).

**Table 4:** Quality Assessment.

Author	Total Sample Size (Group Size)	Risk of Bias Assessment							Details on Needling									Details on Outcome Assessment		
		Random Sequence Generation	Allocation Concealment	Blinding of Participants	Blinding of Outcome Assessment	Incomplete Outcome Data	Selective Reporting	Other Bias	Number of Needles per Session	Points	Depth	Response (Deqi/Twitch)	Stimulation	Needle in Time	Needle (Trademark or Length/Diameter)	Number of Sessions	Frequency/Duration of Treatment	Body Site	Time Point	Tool (Trademark, Detailed Description)
Abuaisha 1998	44	n.c.	n.c.	n.c.	n.c.	+	?	+	x	x	x	-	-	x	-	x	x	x	x	x
Ahn 2007	7 (4/3)	?	?	?	?	-	?	+	x	-	x	x	x	-	-	x	x	x	x	x
Amand 2011	36 (12/12/12)	+	+	+	?	+	?	+	x	x	x	x	x	x	x	x	x	x	x	x
Anderson 1974	30 (10/10/10)	?	+	+	+	+	?	+	x	x	-	-	x	x	x	x	x	x	x	x
Ashton 1984	46:4 (10/13/11/12)	?	?	+	?	+	?	+	x	x	x	-	x	x	x	x	x	x	x	x
Barlas 2000	48(12/12/12/12)	?	?	+	?	?	?	+	x	x	x	-	x	x	x	x	x	x	-	x
Barlas 2006	48 (12/12/12/12)	+	?	+	+	+	?	+	x	x	x	x	x	x	x	x	x	x	x	x
Benoliel 2011	20 (10/10)	?	?	?	?	+	?	+	x	x	-	-	x	x	x	x	x	x	x	x
Berlin	30(10/10/10)	?	?	+	+	+	?	+	x	x	x	x	x	x	x	x	x	x	x	x
Brockhaus 1990	MA trial 40 (25/15)	?	?	+	?	+	?	+	x	x	x	-	x	x	x	x	x	x	x	-
Chae 2006	15	n.c.	n.c.	n.c.	n.c.	+	?	+	x	x	x	-	x	x	x	x	x	x	x	-
Chou Li-Wie 2011	45 (15/15/15)	+	+	+	+	+	?	+	x	x	x	x	x	x	x	x	x	x	x	x
Clark 1974	12 (6/6)	?	?	?	?	+	?	+	x	x	-	x	x	x	-	x	x	x	x	x
Croze 1976	8 (cross over)	?	+	+	?	+	?	?	x	x	x	-	x	x	x	x	x	x	x	x
Day 1975	4	n.c.	n.c.	n.c.	n.c.	+	?	?	x	x	-	-	x	x	-	x	x	x	x	x
Deluze 1992	70 (36/34)	+	+	+	+	+	?	+	x	x	x	x	x	-	x	x	x	x	x	x
Downs 2005	18 (cross over)	?	?	+	?	+	?	+	x	x	-	-	-	x	x	x	x	x	x	x
Edwards 2003	40 (14,13,13)	+	+	-	+	+	?	+	-	x	x	x	x	x	x	x	x	x	x	x
Farber 1997	8	n.c.	n.c.	n.c.	n.c.	+	?	+	x	x	x	x	x	x	x	x	x	x	x	x
Fernande 2010	12 (cross over)	+	+	+	+	+	?	+	x	x	x	x	x	x	x	x	x	x	x	x
Fu 2007	47 (25,22)	?	?	?	?	+	?	?	x	x	x	-	x	x	-	x	x	x	x	x
Galloon 1977	13 (cross over)	?	?	+	?	+	?	?	-	-	-	-	-	-	-	x	x	x	x	x
Goddard 2002	18 (10/8)	+	-	+	+	+	?	?	x	x	x	x	x	x	-	x	x	x	x	-
Harris 2006	65 (12-19)	+	+	+	+	+	?	?	x	x	-	x	x	x	x	x	x	x	x	x
Harris 2008	10 (6/4)	?	?	+	+	?	?	?	-	-	-	-	-	-	-	-	x	x	x	x
He 2004	24 (Aku14/10)	+	?	+	+	+	?	-	x	x	x	-	x	x	x	x	x	x	x	x
Hübscher 2008	22 (7/8/7)	+	+	+	+	+	?	+	x	x	-	x	-	x	x	x	x	x	x	x
Ilbuldu 2004	60∶3 (20)	?	?	+	+	?	?	+	x	x	x	-	-	-	x	x	x	-	x	x
Irnich 2001	177 (56/60/61)	+	+	+	+	+	+	+	-	-	-	-	-	x	-	x	x	x	x	x
Irnich 2003	50 (25/25)	-	-	+	+	+	+	+	x	x	x	x	-	x	-	x	x	x	x	x
Itoh 2011	22 (not indic.)	+	+	?	+	?	?	+	x	x	x	-	x	x	x	x	x	x	x	x
Karst 2000	39 (21/18)	?	?	+	+	?	?	+	x	-	-	-	-	x	x	x	x	x	x	x
Kitade 1979	5 (cross over)	?	?	+	?	+	?	?	x	x	x	x	x	x	x	x	x	x	x	x
Kitade 1981	3 (cross over)	?	?	+	?	+	?	+	x	x	-	-	x	x	-	x	x	-	x	x
Kitade 1988	15(all every int.)	?	?	+	?	+	?	+	x	x	x	-	x	x	x	x	x	-	x	x
Knox 1977	48 (24/24)	?	?	-	-	+	?	+	x	x	x	-	x	x	x	x	x	x	x	x
Knox 1977	48 (24/24)	?	?	-	-	+	?	+	x	x	x	-	x	x	x	x	x	x	x	x
Knox 1979	72 (24/24/24)	?	?	+	+	+	?	+	x	x	x	-	x	x	x	x	x	x	x	x
Knox 1981	40 (20/20)	?	?	-	-	+	?	+	x	x	x	-	x	x	x	x	x	x	x	x
Kong 2005	11 (cross over)	?	?	+	-	+	?	+	x	x	-	x	x	x	x	x	x	x	x	x
Kong 2009	24 (12/12)	?	?	+	?	+	-	+	x	x	x	x	x	x	x	x	x	x	x	x
Kotani 2001	70 (23/23/24)	+	+	+	+	+	?	+	-	x	x	-	x	x	x	x	x	x	x	x
Kumnerddee 2009	18 (9/9)	?	?	-	+	+	?	+	x	-	-	-	-	-	-	x	x	-	-	-
Lang 2010	24 (cross over)	?	?	?	+	+	+	+	x	x	-	x	x	x	x	x	x	x	x	x
Leung 2005	13	n.c.	n.c.	n.c.	n.c.	+	?	+	x	x	x	x	x	x	x	x	x	x	x	x
Leung 2008	16 (cross over)	?	?	?	?	?	?	+	x	x	x	x	x	x	x	x	x	x	x	x
Li 2008	22 (cross over)	?	?	+	+	+	?	+	x	x	x	x	x	x	x	x	x	x	x	x
Li 1983	25 (cross over)	-	-	?	?	+	?	?	x	x	-	x	-	-	-	x	-	x	x	-
Lim 1977	60 (10/10/10/10/10/10)	?	?	?	?	+	?	+	x	x	-	x	x	x	-	x	x	x	x	x
Lin 1981	8 (cross over)	?	?	?	?	+	?	?	x	x	x	x	x	x	x	x	x	x	x	x
List 1993	55 (20/20/15)	?	?	-	?	?	?	+	x	x	-	-	x	x	-	x	x	x	x	x
Lloyd 1976	8	n.c.	n.c.	n.c.	n.c.	+	?	+	x	x	-	-	-	x	x	x	x	x	-	x
Lundeberg 1988	patients 35 (7/7/7/7/7)) healthy controls 15 (3/3/3/3/3/3)	?	?	+	?	+	?	+	x	x	x	x	x	x	x	x	x	x	x	x
Lundeberg 1989	6 (cross over)	-	-	+	?	+	?	?	x	x	x	x	x	x	x	x	x	-	x	x
Lynn 1977	24 (4/4/4/6/6)	-	-	?	?	+	?	+	x	x	-	x	x	x	x	x	x	-	x	x
Ma 2010	43 (15/15/13)	+	?	-	?	+	?	+	-	x	x	x	x	x	x	x	x	-	x	x
Moret 1991	8 (cross over)	?	?	+	?	?	?	-	x	x	-	x	x	x	x	x	x	x	x	x
Nabeta 2002	34 (17/17)	+	+	+	-	+	?	+	-	x	x	x	x	x	x	x	x	x	x	x
Pauser 1975	16 (cross over)	?	?	+	+	?	?	?	-	-	-	-	x	x	-	x	x	-	x	x
Perez-Palomares 2010	122 (67/68)	+	-	?	+	?	?	+	x	-	-	x	x	-	x	x	x	-	x	x
Price 1984	15	n.c.	n.c.	n.c.	n.c.	?	?	+	x	x	x	-	x	x	x	x	x	x	x	x
Schliessbach 2011	45 (cross over)	+	?	+	+	+	?	-	x	x	x	-	x	x	x	x	x	x	x	x
Schliessbach 2012	45 (cross over)	+	?	+	+	+	?	-	x	x	x	-	x	x	x	x	x	x	x	x
Seidel 2002	51 (13/12/13/13)	+	+	+	?	+	?	+	x	x	-	x	x	x	x	x	x	-	-	-
Shen 2007	16 (10/6)	-	-	+	+	+	?	+	x	x	x	-	x	x	x	x	x	x	x	-
Shen 2009	28 (16/12)	+	-	+	+	+	?	+	x	x	x	x	x	x	x	x	x	x	x	x
Shukla 2011	10 (cross over)	?	?	+	?	+	?	+	x	x	x	x	x	x	x	x	x	x	x	x
Singh 2006	21	n.c.	n.c.	n.c.	n.c.	?	?	?	-	-	x	-	-	-	x	x	x	x	x	-
Sprott 2000	20	n.c.	n.c.	n.c.	n.c.	?	?	+	-	-	-	-	-	-	-	x	x	x	x	x
Srbley 2010	40 (20/20)	+	?	+	+	+	?	+	x	x	x	x	x	x	x	x	x	x	x	x
Stern 1977	20 (cross over)	?	?	?	?	+	?	?	x	x	-	-	x	x	x	x	x	x	x	x
Stewart 1977	12 (cross over)	-	-	?	?	+	?	?	x	x	x	-	x	x	-	x	x	x	x	-
Takeda 1994	40 (20)	?	?	+	+	?	?	+	x	x	-	x	x	x	x	x	x	x	x	x
Targino 2008	58 (34/24)	+	?	-	+	+	?	+	x	x	x	x	x	x	x	x	x	x	x	x
Tong 2010	63 (42/21)	+	-	+	?	?	?	+	x	x	x	x	x	x	x	x	x	x	x	x
Ulett 1978	20 (cross over)	?	?	-	-	?	?	?	x	x	-	-	x	x	x	x	x	x	x	x
Umino 1984	10	n.c.	n.c.	n.c.	n.c.	+	?	+	x	x	-	-	x	x	-	x	x	-	x	x
Vincente-Barrero 2012	20 (10/10)	?	?	-	-	?	?	+	-	-	x	-	-	x	x	x	x	x	x	x
Wang 2009	56 (14/14/14/14)	+	-	+	+	+	?	+	x	x	x	x	x	x	x	x	x	x	x	x
Xue 2004	40 (cross over)	+	?	+	+	+	?	+	-	-	-	x	x	x	-	x	x	x	x	-
Yoon 1986	9 (cross over)	?	?	?	?	+	?	?	x	-	-	x	x	-	-	-	-	x	-	-
Zaslawski 2003	13 (cross over)	?	+	+	+	+	?	?	x	x	x	-	x	x	x	x	x	x	x	x
Zhang 2003	8 (cross over)	-	-	+	?	+	?	+	x	x	-	-	x	x	-	x	x	x	x	x
Zhang 2009	53 (28/25)	+	+	+	+	+	?	+	x	x	x	x	x	x	x	x	x	x	x	x
Zhu 2002	29 (cross over)	?	?	+	+	+	?	+	x	-	x	x	x	x	x	x	x	x	x	-

Within the risk of bias assessment ‘+’ indicated ‘low risk of bias, ‘-’indicates ‘high risk of bias’ and ‘?’ indicates ‘unclear risk of bias’. Studies lacking a control group are marked with ‘n.c.’ indicating ‘high risk of bias’. Reporting of details about the intervention and assessment of sensory thresholds are indicated as in accordance with the STRICA guidelines with ‘x’ and as missing with ‘-’. In addition, the total sample size as well as the number of subjects in the respective groups is displayed in parenthesis.

**Figure 2:**
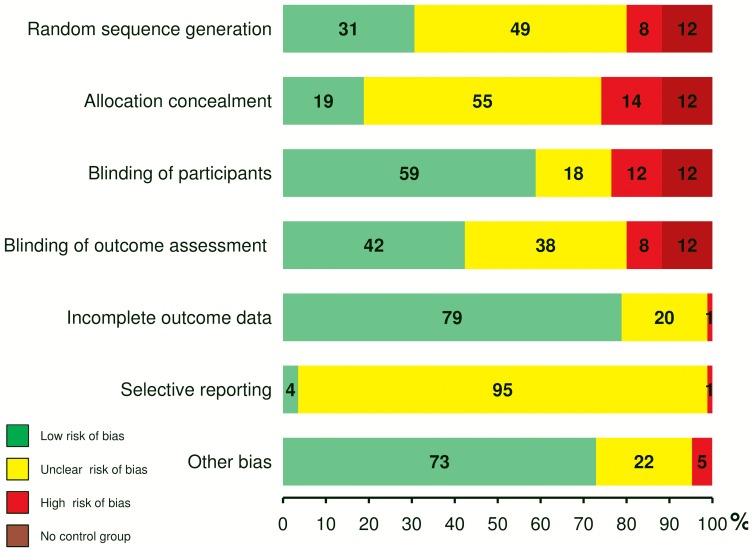
Risk of Bias Assessment. Results of the risk of bias assessment as depicted in table 4 are summarized. Percentages of studies with ‘low risk of bias’, ‘high risk of bias’, or ‘unclear risk of bias’ are illustrated for each item of the Cochrane Collaboration's tool.

Results of the risk of bias assessment are presented in table 4 and in summary in fig. 2. Only six studies (7.1%) were rated as ‘low risk of bias’ in six or more items of the Cochrane risk of bias tool [33], [37], [40], [47], [49], [62]. A judgment as ‘high risk of bias’ resulted most frequently from methodological shortcomings within the randomization process and the choice of comparison groups not allowing for blinding of participants (8.2% random sequence generation, 14.1% allocation concealment, 11.8% blinding of participants). Blinding of the outcome assessment was assured in less than half of the included studies (42.4%). The overall large proportions of studies with ‘unclear risk of bias’ reflects the low quality of reporting. In contrary, overall reporting of assessments of sensory thresholds was good and treatment regimens were reported in a mediocre manner (table 4). In 23 of the included publications (27.1%), information was missing for three or more items of the STRICTA guidelines [21]. About a quarter of all studies (24.7%) met all of the nine treatment related items. Threshold measurements were well described in 62 studies (72.9%), but especially combinations of different threshold assessments lacked standardization. Among the eight studies (9.4%), that evaluated acupuncture evoked changes in more than one modality of sensory perception, there was a single study which applied a comprehensive and standardized test battery for sensory testing [100].

### Comparison of Verum Acupuncture to Inert or Sham-Control Procedures

Nineteen studies (22.4%) compared acupuncture to an inert control such as a placebo pill [26], [42], a waiting list [38], [62], [68], or a period of rest [24], [25], [27], [30], [35], [47], [51], [53], [56]–[59], [85], [99]. In 12 of these studies, acupuncture had a greater effect than the inert control procedure [24]–[26], [30], [38], [51], [53], [57], [58], [62], [68], [85], while results of six studies indicate no or only a minor change of the respective sensory threshold in any of the study groups [27], [42], [47], [56], [59], [99]. Results of one study were unclear . One article reported a significant change of sensory thresholds after an inert control procedure [24]. All four studies evaluating acupuncture as an add-on treatment reported a significant additional effect [38], [46], [70], [87].

In 48 studies (56.5%) one or several types of sham acupuncture treatments were used as controls. In the following, the term “sham” is used according to the description of the respective publication. Sham interventions were needling at non-acupuncture points with stimulation (17 out of 48; 35.4%) [24], [25], [30], [31], [35], [42]–[44], [50], [53], [62], [73], [83]–[85], [94], [95], without or with minimal stimulation (10 out of 48; 20.8%) [22], [27], [37], [44], [46], [47], [57], [86], [93], [95], minimal or no intensity needle stimulation at classical acupuncture points (10 out of 48; 20.8%) [40], [44], [66], [80], [88], [92], [95], [97], [103], [104], non-penetrating placebo needles (12 out of 48; 25%) [28], [33], [45], [52], [60], [61], [72], [76], [78], [79], [98], [99], or treatment with an inactive laser pen (5 out of 48; 10.4%) [31], [48], [49], [77], [95]. In 25 of these 48 studies (52.1%) the effect on sensory thresholds was significantly larger in the verum than in the sham acupuncture group [25], [28], [31], [33], [37], [40], [46], [50], [52], [57], [60], [62], [72], [76], [78]–[80], [83]–[85], [88], [92], [93], [95], [97]. In two studies (4.2%), it was shown descriptively that verum acupuncture was superior to sham acupuncture, but no statistical analysis was provided [53], [94]. In nine studies (18.8%) both, verum and sham acupuncture, had a significant effect on the respective sensory threshold [43], [61], [62], [66], [73], [76], [77], [86], [98]. Among these, two studies also found verum acupuncture to cause significantly larger threshold changes than sham interventions [62], [76]. In nine other studies (18.8%) no effect was observed neither after verum nor after sham acupuncture [22], [27], [42], [47]–[49], [99], [103], [104], and the results of three studies (6.3%) were rated as unclear [35], [44], [45]. None of the twelve studies including both control modalities found significant differences between the sham and the inert control group [24], [25], [27], [30], [35], [42], [47], [53], [57], [62], [85], [99], although in three of these studies, the pre-post comparison indicates larger effects in the sham than in the inert control group [24], [30], [53], [85].

### Heat Pain Threshold (HPT)

All 26 studies (30.6%) that assessed whether acupuncture had an effect on the HPT evaluated immediate treatment effects. Nineteen of these studies (73.1%) showed that HPT was elevated through acupuncture. In five articles (19.2%), no statistical analysis was provided. Nevertheless, in three cases we agree with a positive rating of the results because of the following reasons. In two studies case numbers were too low to allow for statistical calculations, but HPT elevations following acupuncture were observed in all subjects [53], [54]. In another study, two thirds of all subjects (6 out of 9, 66.6%) showed an elevated HPT after EA, but not after stimulation at non-acupuncture points [94]. In contrast, the results of two studies which claim to indicate an effect of acupuncture on the HPT were rated as unclear, because mean HPT changes were not calculated [55], or data were depicted in a graph only [35].

All but two studies (92.3%) assessed the effect of acupuncture on the HPT in healthy volunteers. Seventeen of these studies (70.8%) focused on the effect of EA only [30], [34], [36], [42], [53]–[55], [61], [66], [69], [80], [85], [90], [94], [101], [102], [105], five studies (20.8%) on the effect of MA only [31], [32], [35], [67], [99], and two studies (8.3%) evaluated both treatment methods . Of the 19 studies evaluating EA, 15 (78.9%) indicated an elevation of the HPT [30], [34], [53], [54], [60], [61], [66], [80], [85], [90], [94], [100]–[102], [105] such as did five of seven (71.4%) MA studies [31], [32], [60], [67], [100].

Price et al. assessed low back pain patients and found a significant effect of EA on the HPT [75]. Lundeberg et al., however, did not find any effect of MA or EA on the HPT neither in patients suffering from sinus pain nor in healthy volunteers [104].

The methods used to determine the HPT varied largely, and some studies evaluated several measures. In 16 out of 26 studies (61.5%), the HPT was defined as the time period that subjects were able to tolerate a defined heat stimulus. Eleven of these studies showed a positive outcome. Defined heat stimuli were produced by a projection lamp held on blackened skin [30], [34]–[36], [42], [53]–[55], [66], [69], [85], [90], by a hot plate [32], [67], or by a thermode containing a peltier element [31]. In one study no information was given about the method used for the application of heat stimuli [94]. In six studies (23.1%) the HPT was defined as the temperature that was first experienced as painful when increasing heat was applied with a thermal sensory analyzer [99]–[102], [104], [105]. Results of three of these studies (50%) indicated an effect of acupuncture on the HPT [100], [102], [105]. Six of the 26 studies (23.1%) assessed the pain intensity evoked by a heat stimuli applied with a thermode [60], [61], [75], [80], [101], [102]. All six found a reduction of the pain intensity after acupuncture.

### Cold Pain Threshold (CPT)

Of the 85 included studies, 17 (20%) addressed the effect of acupuncture on the CPT. In 11 of these 17 studies (64.7%), subjects were less sensitive to cold pain after than before acupuncture. All studies focused on the immediate effect after one single acupuncture treatment. Sixteen studies (94.1%) were conducted with healthy volunteers and one with chronic pain patients. When assessing healthy volunteers, eight studies (50%) showed a significant change of the CPT through EA [25], [57], [58], [71], [84], [92], [97], [105], while four studies (25%) found no effect of EA [56], [59], [101], [102]. Three studies (18.8%) conducted with healthy volunteers investigated the effect of MA. Two of these showed a desensitizing effect [24], [26] and one no effect of acupuncture [99]. Lang et al. assessed both, MA and EA, and did not find any effect on the CPT [100]. Ulett et al. showed that sensitivity to cold pain of chronic pain patients was decreased after EA [89].

Different methods were used for the CPT measurements. In ten studies (58.8%), the CPT was assessed by immersion of the subjects' fingers or forearm into ice-water and documenting either the time until subjects withdrew their limb [24], [26], [71] or the pain intensity that was experienced during a defined period of immersion into ice water [25], [56]–[59], [84], [89]. Eight of these ten studies (80%) showed a significant reduction of the CPT after acupuncture. In one study, contact to an ice cold cylinder was tolerated longer after than before acupuncture [105]. In six studies (35.3%), cold stimuli were applied with a thermode including a peltier element and either the intensity of the pain evoked by defined cold stimuli [97] or the temperature that was considered painful [92], [99]–[102] was evaluated. Four of the latter showed no changes of the CPT [99]–[102].

### Thermal Detection Thresholds

#### Warm Detection Threshold (WDT)

The effect of acupuncture on the WDT was investigated in six studies (7.1%) with five evaluating the immediate effect in healthy subjects. Among these, significant changes of the WDT were shown in one study after MA [29] and in two studies after EA [101], [102]. No effect of acupuncture in healthy volunteers was reported in two studies, one on MA [99] and one assessing both MA and EA [100]. Ahn et al. included patients suffering from painful diabetic neuropathy (PDN) and measured the WDT before and after a series of treatments [106]. The results of this study are rated as unclear due to the lack of a statistical analysis and the fact that data are depicted in a graph only.

#### Cold Detection Threshold (CDT)

Five of the six studies that investigated the effect of acupuncture on the WDT also assessed changes in the CDT after acupuncture. Correspondingly, results of one study assessing the CDT in PDN patients were rated as unclear [106]. Of the four remaining studies, three found no change of the CDT after MA [99], [100] or EA [101], and two found a decrease of the CDT after EA [100], [102].

All but one study, for which the measuring tool was not described, used a thermode including a peltier element to assess the WDT and the CDT.

#### Thermal Sensory Limen (TSL)

Three of the included acupuncture studies (3.5%) used the TSL as an outcome measure. All three investigated immediate treatment effects. In two studies by Lundeberg et al., the TSL was found unchanged after EA as well as after MA in healthy volunteers and in patients suffering from sinus pain [103], [104]. In contrast, Lang et al. found a significant elevation of the TSL of up to 3°C through EA but not through MA in healthy volunteers [100]. In all studies, TSL assessments were performed by using a sensory thermal analyzer. The thermode applied on the subjects' skin increases or decreases its temperature. As soon as the subject indicates the feeling of cool or warm, a switch towards the opposite direction of the temperature change is induced.

### Pressure Pain Threshold (PPT)

Almost half of the articles (42 out of 85, 49.4%) included in this review describe changes of the PPT through acupuncture.

#### PPT assessed in Healthy Subjects

Seven of these 42 studies (16.7%) evaluated the immediate effect of acupuncture on the PPT in healthy subjects. All showed a statistically significant elevation of the PPT through acupuncture. Two studies assessed the effect of EA only [28], [39], three studies solely the effect of MA [65], [95], [98], and two the effect of both, MA and EA . Either a manual algometer [39], [65], [95], [100] according to Fischer et al. [107] or an electronic algometer [28], [76], [98] was used as measuring tool (tip size 1 cm^2^). Changes ranged from 59 to 392 kPa [28], [39], [76], [98], [100] or from 10% to 27%, respectively [65], [95].

#### PPT assessed in Pain Disorders

The assessment of the PPT was widely used (35 out of 42, 83.3%) in order to evaluate the effectiveness of acupuncture in reducing hyperalgesia in pain disorders. Most common conditions under investigation were myofascial pain, associated with the occurrence of MTrPs (8 out 35, 22.9%) [33], [38], [41], [48], [50], [63], [70], [83], fibromyalgia (7 out of 35, 20%) [37], [44], [45], [72], [81], [82], [87], chronic neck and back pain (5 out of 35, 14.3%) [22], [46], [49], [74], [77], temporomandibular disorder (5 out of 35, 14.3%) [40], [43], [68], [79], [91], and experimentally induced delayed-onset muscle soreness (DOMS; 4 out of 35, 11.4%) [27], [47], [51], [78]. PPT was also used as an outcome measure for the treatment effect of acupuncture in tension type headache [52], [93], osteoarthritis of the knee [86], abdominal scar pain [62], plantar fasciitis [96], and syringomyelia [64].

Ten of the 35 patient studies (28.6%) evaluated the immediate effect of one single acupuncture treatment. Nine of these reported the PPT to be significantly elevated [33], [40], [41], [43], [51], [64], [78], [79], [83], and one study found no significant PPT increase [50]. Among the 26 studies (74.3%) that assessed the effectiveness of an acupuncture series, 18 (69.2%) showed a PPT increase after treatment [37], [38], [46], [50], [52], [62], [63], [68], [70], [72], [74], [77], [81], [82], [86], [87], [91], [93] while six studies (23.1%) observed no effect of acupuncture on the PPT [22], [27], [47]–[49], [96]. The outcome of two studies was rated as unclear, because the analysis was performed by combining data of all groups [44], [45]. Although no statistics were provided, the outcome of two other studies was rated as positive. Singh et al. found that, after a MA treatment of two months, the majority of fibromyalgia patients felt less pain, although greater pressure than at baseline was applied [81]. Perez-Palomarez et al. included a large number of patients and showed a prominent mean increase of the PPT (74.5 kPa–202.0 kPa) after DN and EA [74].

The effect of EA on the PPT was evaluated in four out of the 35 studies (11.4%) conducted with patients [37], [64], [74], [93]. All four showed an increase of the PPT after EA. MA was investigated in 22 studies (62.9%) of which 17 (77.3%) revealed an increase of the PPT . In two of these studies, MA was applied as an add-on treatment to stretching [70] or tricyclic antidepressants and exercise [87] for myofascial pain or fibromyalgia, respectively. Seven of the 35 studies (20.0%) investigated the effects of DN in the treatment of myofascial pain. DN was shown to be effective in increasing the PPT in six studies [33], [38], [40], [41], [74], [83], but one study found no effect of DN on the PPT at MTrPs of the neck [48]. Four studies used a combination of either EA and MA [22], [46], [68] or of MA and DN [49]. Two of these studies showed a change of the PPT [46], [68] and two no treatment effect [22], [49]. The observed PPT changes varied largely between studies (22.5 kPa to 245.2 kPa) and tended to increase during follow up if assessed.

Three studies also assessed the pressure pain tolerance (PPTo) which was defined as the time subjects tolerated painful pressure [45], [64], [74]. Two studies showed the PPTo to be elevated after EA [64] and DN [74], respectively. Results of one study on MA were rated as unclear [44].

Study outcome was not associated with a certain methodology used for the PPT assessment. One study did not provide details about the measuring tool (spring roller) [64]. In all other studies (34 out of 35, 97.1%) algometers with different tip sizes (between 0.28 and 3.14 cm^2^) were used to determine the PPT. In 23 studies, algometers were equipped with a rubber tip of 1 cm^2^ or larger [27], [33], [37], [38], [40], [41], [44]–[48], [50], [62], [68], [70], [74], [79], [81], [82], [86], [87], [91], [96], while in five studies tips of smaller sizes were used [49], [51], [52], [72], [83], [93]. In five articles no information was given about the characteristics of the algometer [22], [43], [63], [77], [78].

#### Meta-Analysis

**Figure 3:**
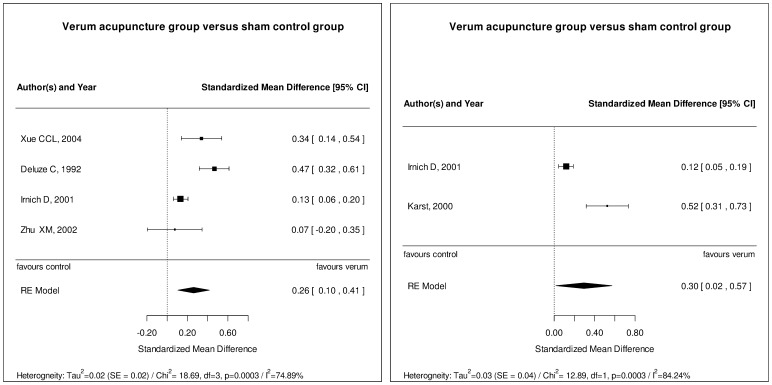
Meta-Analysis. Considering the previously defined quality criteria (see methods) as well clinical homogeneity according to the investigators accordance, five studies on the effect of acupuncture on the PPT conducted with patients were combined in two meta-analyses. The short-term effect on the PPT directly after a series of acupuncture sessions as assessed in four studies (A) was found to be significant but small. Two studies were analyzed regarding long-term effects of acupuncture on the PPT (B) which was also found to be significant but small. Heterogeneity was found to be substantial in both analyses.

Considering the previously defined quality criteria and clinical homogeneity, only studies investigating the effect of acupuncture on the PPT were eligible for meta-analyses. Four studies could be combined in an analysis regarding the immediate treatment effect [22], [37], [49], [93], and two studies were analyzed regarding long-term effects of acupuncture in pain conditions [49], [52]. The results of these meta-analyses confirm our descriptive findings. The effect of acupuncture directly after a series of treatments (fig. 3A) and at follow up time points (6 weeks [52] and 3 months [49]; fig. 3B) was shown to be significant (p<0.05). However, effects were small (SMD 0.26 [95% CI: 0.1; 0.41] for immediate effects, SMD 0.3 [95% CI: 0.02; 0.52] for long-term effects) and heterogeneity was found to be substantial.

### Mechanical Pain Threshold/Sensitivity (MPT/MPS)

Three of the 85 articles (3.5%) included in this review investigated the effect of acupuncture on the MPT and/or the MPS. All three studies were conducted with healthy volunteers assessing the MPT/MPS before and immediately after a single acupuncture treatment. All showed a desensitizing effect of EA as well as of MA [73], [100], [105]. Just Lang et al., who assessed both, the MPT and the MPS, did not find an effect of MA on the MPT [100]. However, different measuring tools were used; scaled forces applied with pin-pricks [100], increasing pressure applied by a blunt needle [73], or gauged forceps [105].

### Mechanical Detection Threshold

We identified two acupuncture studies (2.4%), in which the MDT was one of the outcome measures. Both studies were conducted with healthy volunteers, used Von Frey Filaments in order to evaluate the MDT, and focused on immediate effects of acupuncture; one on EA [102] and one on both EA and MA [100]. Results of these two studies indicate that neither EA nor MA has an impact on the MDT in healthy subjects.

### Vibration Detection Threshold (VDT)

Four articles (4.7%) describe the effect of acupuncture on the VDT. In two studies the impact of a MA treatment series on the VDT in patients suffering from diabetic peripheral neuropathy was explored. Tong et al. noted an improvement of the ability to detect vibration [88] while Abuaisha et al. found no changes of the VDT after MA [23], [88]. Lang et al. and Lundeberg et al. reported that a single application of neither EA nor MA had an effect on the VDT in healthy volunteers [100], [103]. Vibration stimuli were applied with a Rydel-Seiffer tuning fork [100] or an electromechanical device [23], [88], [103].

### Effect of Needle Location relative to the Sites of Measurements

Twenty (23.5%) studies compared ipsi- to contralateral and/or close to distant needling either in healthy subjects (15 out of 20; 75%) or in pain patients (5 out of 20; 25%). Of the ten studies comparing close to distant needling, eight (80%) showed a larger increase of at least one sensory threshold close to the needling location [35], [51], [53], [67], [83], [101], [102], [105]. In six out of eleven studies (54.5%), threshold changes were more pronounced after ipsi- than after contralateral needling [25], [34], [67], [100]–[102]. In one high quality study bilateral needle placement was superior to unilateral needling [65], and results of three studies suggest that needle stimulation at LI 4 is more effective in changing pain thresholds than needling at other acupuncture points [39], [53], [65]. Four studies (20%) found significant effects of acupuncture on sensory perception independent of the needle location [28], [60], [66], [75], [95], and in two studies no change of any sensory threshold was observed [103], [104].

### Responder versus Non-responders

Six of all included studies (7.1%) distinguished between subjects that responded to acupuncture and those who did not [32], [35], [42], [55], [60], [94]. All of these studies assessed the HPT in healthy volunteers and all were conducted with relatively small case numbers (11.8±3.0). Proportions of responders ranged from one third to two thirds. Chae et al. found genetic difference between acupuncture responders and non-responders [32], but their results have not been reproduced by further investigations. Furthermore, the role of hypnotic susceptibility in responsiveness to acupuncture was investigated in three studies, with contradictory results [56], [58], [89]. The influence of expectancy was found to be substantial in two studies [57], [61]. Knox et al. found no effect of EA neither in oriental nor occidental subjects [59].

## Discussion

### Result Interpretation

Our results revealed that in 76.5% of 85 eligible studies at least one sensory threshold was changed after acupuncture, indicating an activation of neuromodulatory mechanisms. However, results displayed substantial heterogeneity, which is illustrated for the PPT by results of the meta-analyses (fig. 3).

Over half of the sham-controlled studies found larger effects in the verum group than in the sham group. However, a quarter of these studies found significant threshold changes also after sham acupuncture, while only one of the included articles reported changes of sensory thresholds after an inert control procedure. These facts go in line with the previously drawn conclusion that there are effects specific to acupuncture, but that sham acupuncture may cause physiological reactions exceeding pure placebo effects [6], [108], [109]. It can be assumed that this in part explains the clinical effects of sham acupuncture interventions observed in acupuncture randomized controlled trials.

Most studies conducted with patients used the PPT as an outcome measure. This reflects the frequent use of acupuncture in clinical practice for treating pain conditions e.g. musculoskeletal disorders, in which the PPT correlates well with clinical status [110]. More than 80% of the studies – i.e. 27 out of 35 clinical studies and all seven studies conducted with healthy volunteers – showed that acupuncture reduced pain evoked by blunt pressure which is mainly mediated by deep tissue nociceptors (Aδ- and C-fibers) [110]. PPT reductions of up to 245.2 kPa as observed in some studies can be interpreted as clinical relevant. In addition, results of two meta-analyses show significant short- and long-term effects of acupuncture on the PPT in pain conditions (fig. 3). Thus, these findings provide a physiological basis for the growing body of evidence for the effectiveness of acupuncture in locomotor conditions associated with tenderness [111]–[113].

Pain thresholds were elevated after acupuncture also when painful pressure was exerted on a rather small skin area (≤1 cm^2^) and when pin-prick like stimuli (MPS and MPT) were applied. This finding is derived from few studies, but suggests that acupuncture also affects mechanical pain evoked by punctate objects which is primarily mediated by intra-epidermal nociceptors (mainly A–δ fibers) [114].

Studies investigating whether thermal pain is reduced through acupuncture are abundant but almost exclusively conducted with healthy volunteers. Results of such investigations are more ambiguous than data of included studies on changes of the PPT, MPT and MPS. After acupuncture, sensitivity to painful heat was reduced in 19 out of 27 studies, while sensitivity to painful cold was reduced in 11 out of 17 experiments. The transmission of heat pain is mainly evoked by C-fiber mechano-heat nociceptors (CMH) responding to heat stimuli ranging from 41°C to 49°C [115] and is linked to the capsaicin sensitive vanilloid receptor VR1 which is also found in type II Aδ-nociceptors [116]. In contrast, the transmission of cold pain is mediated by both cold sensitive C- and Aδ-fibers [117], [118] which are insensitive to vanilloid compounds [119]. Our results suggest that both types of nociception are likely to be affected by acupuncture in healthy subjects. Reasons for the heterogeneity of data might partly be explained by methodological issues. Studies in which painful cold was applied through a thermode found changes of the CPT less frequently (two versus four) than other studies (eight versus two), in which e.g. the subject's hand or arm was immersed into ice-water. It is conceivable that differences in study outcome are related to the size of the skin area to which cold stimuli were applied or to the intensity of these stimuli (see 4.2. *Limitations* for further discussion).

In contrast to pain perception, data on the effect of acupuncture on sensory detection are sparse. The ability to detect temperature changes (WDT, CDT, TSL) was reduced after acupuncture in half of all experiments. Mechanical detection (VDT and MDT), in contrast, was not affected by acupuncture in five out of six trials. These findings provide a first hint that acupuncture might not affect mechanical detection which is mainly mediated by Aβ-fiber signaling [120], [121], while the influence on thermal detection which is linked to signaling of warm sensitive C-fibers or cold sensitive Aδ- and C-fibers [122], [123] remains unclear.

Another important finding is that ipsilateral needling and needling close to the measure sites were found to exhibit stronger effects on sensory thresholds than needling at contralateral or remote body sites, respectively. This underscores the importance of local mechanisms such as the release of neuromodulators at the needling site [14] and spinal mechanisms such as segmental inhibition [11]. It is supposed, that activation of A-fiber afferents results in the activation of spinal inhibitory interneurons, those achieving primary analgesia within the same segment [124], [125]. Nevertheless, more studies adopting a sophisticated selection of measure sites in combination with imaging studies are needed to clearly differentiate between local, spinal and supraspinal mechanisms of acupuncture. In contrast, it seems to be of minor importance whether the needle is stimulated electrically or manually.

### Limitations

The overall poor study quality (fig. 2) and the consequently low number of studies included in the meta-analyses are the major limitations to our findings. However, limitations resulting from the quality assessment itself need to be taken into account. Many publications were characterized by poor reporting which renders an estimation of the real number of high quality studies/studies with low risk of bias impossible. In particular details of blinding and randomization procedures as well as treatment regimens were often missing. Second, the STRICTA guidelines provide an essential tool for assuring the quality of reporting in acupuncture trials. Nevertheless, it bears the striking disadvantage that the quality of reporting of treatment related items might be underestimated in studies investigating the effectiveness of individualized acupuncture regimens since precise instructions for such studies are missing.

A further limitation comes with the methodology of QST itself. Although when performed with standardized methods, QST involves subjective ratings of perceptions and, therefore, is susceptible to bias due to psychological factors such as expectation and conditioning [126]. These should be assessed and/or controlled for [127]. Comparability of studies was also limited due to methodological variability with regard to treatment, study population, and outcome assessment. Appropriate selection of the acupuncture points needled, number of needles and stimulation technique are, according to the traditional concept of acupuncture, crucial for achieving an optimal treatment effect. These concepts are in part supported by research findings. For example, current evidence suggests an association between the number of needles applied and the clinical outcome [128], but overall there is still no consensus within the scientific community about how to appraise these parameters correctly. The same holds for the socio-cultural background of the study population. Previous acupuncture experience and social valuation of acupuncture is very likely to have an impact on treatment outcome; likewise to any other treatment. Yet, there is very little emphasis on sociocultural aspects in acupuncture research. There were striking differences between acupuncture interventions applied in the included studies and, although not reported in detail, very probably also between the different study populations. However, due to the still poor knowledge about the impact of these factors, there are no guidelines on how to appraise them in a review apart from pure reporting as performed in table 1 and 2.

Additionally, study outcome might in part be influenced by the measuring tool. Investigations comparing different methods evaluating the same sensory thresholds are missing. It also remains unclear to what extent the test stimuli might interfere with the treatment effect. For example, strong noxious stimulation is known to activate aspects of the endogenous pain-modulating network [129]–[131]; a phenomenon known as counter irritation. It is postulated that the main underlying mechanisms namely DNIC is also involved in the analgesic effect of acupuncture [98], [132]–[134]. This is however controverted by recent work and needs to be further explored [98], [134].

Furthermore, the interpretation of the clinical relevance of the effect of acupuncture is limited by the fact that studies - besides those assessing the PPT – were almost exclusively conducted with healthy volunteers. There is also limited amount of data encoding the effect of acupuncture on stimuli above pain thresholds, which have been shown to activate other nerve fibers than stimuli around the pain threshold [135].

### Future Perspectives

Recently, efforts are increasing to comprehensively characterize painful conditions by means of QST [136]–[139]. Attempts are made to classify patients on the basis of symptoms, signs, or patterns of somatosensory abnormalities [139]–[141]. Those might reflect the underlying pathological mechanisms [142] and might, therefore, be related to different treatment responses [137], [143]. Such subgroup analysis should also be adopted in acupuncture research since responsiveness to acupuncture varies largely between individuals. This inter-individual variability in response to acupuncture was also demonstrated by studies included in this review, but proposed hypotheses explaining this phenomenon have not been verified. Comprehensive QST assessments applied in large scale studies might provide hints about whether individual patterns of somatosensory alterations might be of predictive value for the effectiveness of acupuncture. It is striking that only one study applied a comprehensive, standardized QST test battery [100].

Comprehensive QST assessments might, furthermore, provide information about the extent to which signs of peripheral and central sensitization may be reduced by acupuncture. For example, it has not yet been subject to clinical trials whether thermal and mechanical hypaesthesia or increased wind-up, which can be associated with chronic pain conditions [144]–[146], might vanish along with clinical improvement after an acupuncture therapy.

### Conclusion

This review provides evidence for the effect of acupuncture on sensory perception, especially pain perception. Most compelling evidence supports the reduction of pain evoked by blunt pressure through acupuncture; particularly as a measure of tenderness in pain conditions. Moderate evidence was found that acupuncture reduces the perception of noxious heat or cold. The outcome of these studies seemed to depend on measuring methods. Little but consistent evidence was found that pin-prick like pain (MPT, MPS) is reduced but that mechanical detection (VDT, MDT) is not influenced by acupuncture. No conclusions can be drawn about whether acupuncture affects the ability to detect temperature changes.

Sham-acupuncture approaches can evoke such effects as well. Thus, it is questionable to classify them as pure placebos, a fact that needs to be taken into account when conducting and interpreting acupuncture studies.

Data support the importance of local and spinal mechanisms involved in the neurophysiological effect of acupuncture. More high quality studies are needed to characterize the effect of acupuncture on the whole sensory profile by means of comprehensive QST assessments. In addition, investigating defined patient populations accordingly might also clarify whether certain characteristics of the somatosensory profile are of predictive value for the analgesic effect of acupuncture and to which extent pathologic alterations of sensory perception vanish along with clinical improvement achieved by acupuncture.

## Supporting Information

S1 Table **PRISMA Checklist.** Overview of the reporting items in accordance with the PRISMA statement
(DOC)
